# Robotic Versus Laparoscopic Liver Resection in Various Settings: An International Multicenter Propensity Score Matched Study of 10.075 Patients

**DOI:** 10.1097/SLA.0000000000006267

**Published:** 2024-03-14

**Authors:** Jasper P. Sijberden, Tijs J. Hoogteijling, Davit Aghayan, Francesca Ratti, Ek-Khoon Tan, Victoria Morrison-Jones, Jacopo Lanari, Louis Haentjens, Kongyuan Wei, Stylianos Tzedakis, John Martinie, Daniel Osei Bordom, Giuseppe Zimmitti, Kaitlyn Crespo, Paolo Magistri, Nadia Russolillo, Simone Conci, Burak Görgec, Andrea Benedetti Cacciaguerra, Daniel D’Souza, Gabriel Zozaya, Cèlia Caula, David Geller, Ricardo Robles Campos, Roland Croner, Shafiq Rehman, Elio Jovine, Mikhail Efanov, Adnan Alseidi, Riccardo Memeo, Ibrahim Dagher, Felice Giuliante, Ernesto Sparrelid, Jawad Ahmad, Tom Gallagher, Moritz Schmelzle, Rutger-Jan Swijnenburg, Åsmund Avdem Fretland, Federica Cipriani, Ye-Xin Koh, Steven White, Santi Lopez Ben, Fernando Rotellar, Pablo E. Serrano, Marco Vivarelli, Andrea Ruzzenente, Alessandro Ferrero, Fabrizio Di Benedetto, Marc G. Besselink, Iswanto Sucandy, Robert P. Sutcliffe, Dionisios Vrochides, David Fuks, Rong Liu, Mathieu D’Hondt, Umberto Cillo, John N. Primrose, Brian K.P. Goh, Luca A. Aldrighetti, Bjørn Edwin, Mohammad Abu Hilal

**Affiliations:** * Department of Surgery, Fondazione Poliambulanza Istituto Ospedaliero, Brescia, Italy; † Department of Surgery, Amsterdam UMC Location University of Amsterdam, Amsterdam, the Netherlands; ‡ Cancer Center Amsterdam, Amsterdam, the Netherlands; § The Intervention Centre and Department of HPB Surgery, Oslo University Hospital and Institute of Medicine, University of Oslo, Oslo, Norway; ∥ Department of Surgery, Ringerike Hospital, Vestre Viken Hospital Trust, Drammen, Norway; ¶ Hepatobiliary Surgery Division, IRCCS San Raffaele Hospital, Milan, Italy; # Vita-Salute San Raffaele University, Milan, Italy; ** Department of Hepatopancreatobiliary and Transplant Surgery, Singapore General Hospital and National Cancer Centre Singapore, Singapore; †† Department of Surgery, University Hospital Southampton NHS Foundation Trust, Southampton, UK; ‡‡ Department of Surgical, Oncological and Gastroenterological Sciences, General Surgery 2, Hepatopancreatobiliary Surgery and Liver Transplantation, Padua University Hospital, Padua, Italy; §§ Department of Digestive and Hepatobiliary/Pancreatic Surgery, Groeninge Hospital, Kortrijk, Belgium; ∥∥ Faculty of Hepatopancreatobiliary Surgery, the First Medical Center of Chinese People’s Liberation Army (PLA) General Hospital, Beijing, China; ¶¶ Department of Digestive, Oncologic and Metabolic Surgery, Institut Mutualiste Montsouris, Université Paris Descartes, Paris, France; ## Department of Surgery, Division of HPB Surgery, Carolinas Medical Center, Atrium Health, Charlotte, NC; *** Liver Unit, Queen Elizabeth Hospital, Birmingham, UK; ††† Digestive Health Institute, AdventHealth Tampa, Tampa, FL; ‡‡‡ Hepato-Pancreato-Biliary Surgery and Liver Transplantation Unit, University of Modena and Reggio Emilia, Modena, Italy; §§§ Department of General and Oncological Surgery, Umberto I Mauriziano Hospital, Largo Turati, Turin, Italy; ∥∥∥ Department of Surgery, University of Verona, Verona, Italy; ¶¶¶ Department of Experimental and Clinical Medicine, Hepatobiliary and Abdominal Transplantation Surgery, Riuniti Hospital, Polytechnic University of Marche, Ancona, Italy; ### Department of Surgery, McMaster University, Hamilton, Ontario, Canada; **** Department of Surgery, HPB and Liver Transplantation Unit, University Clinic, Universidad de Navarra, Institute of Health Research of Navarra (IdisNA), Pamplona, Spain; †††† Servei de Cirurgia General i Digestiva, Hospital Doctor Josep Trueta de Girona, Girona, Catalonia, Spain; ‡‡‡‡ Department of Surgery, Division of Hepatobiliary and Pancreatic Surgery, University of Pittsburgh Medical Center, Pittsburgh, PA; §§§§ Department of General, Visceral and Transplantation Surgery, Clinic and University Hospital Virgen de la Arrixaca, IMIB-ARRIXACA, El Palmar, Murcia, Spain; ∥∥∥∥ Department of General, Visceral, Vascular and Transplant Surgery, University Hospital Magdeburg, Magdeburg, Germany; ¶¶¶¶ Department of Surgery, Newcastle upon Tyne Hospitals NHS Foundation Trust, Newcastle upon Tyne, UK; #### Department of Surgery, IRCCS Azienda Ospedaliero-Universitaria di Bologna, Bologna, Italy; ***** Department of Hepato-Pancreato-Biliary Surgery, Moscow Clinical Scientific Center, Moscow, Russia; ††††† Department of Surgery, Virginia Mason Medical Center, Seattle, WA; ‡‡‡‡‡ Department of Surgery, University of California San Francisco, CA; §§§§§ Hepato-Pancreato-Biliary Surgery Unit, Miulli Hospital, Acquaviva delle Fonti, Bari, Italy; ∥∥∥∥∥ Department of Digestive Minimally Invasive Surgery, Antoine Béclère Hospital, Paris, France; ¶¶¶¶¶ Chirurgia Epatobiliare, Università Cattolica del Sacro Cuore-IRCCS, Rome, Italy; ##### Department for Clinical Science, Division of Surgery, Intervention and Technology (CLINTEC), Karolinska Institutet, Karolinska University Hospital, Stockholm, Sweden; ****** University Hospitals Coventry and Warwickshire, Clifford Bridges Road, Coventry, UK; †††††† St. Vincent’s University Hospital, Elm Park, Dublin, Ireland; ‡‡‡‡‡‡ Department of General, Visceral and Transplant Surgery, Medizinische Hochschule Hannover, Hannover, Germany; §§§§§§ Department of Surgery, Division of Abdominal Transplantation, Carolinas Medical Center, Atrium Health, Charlotte, NC; ∥∥∥∥∥∥ Surgery Academic Clinical Program, Duke-National University of Singapore Medical School, Singapore, Singapore

**Keywords:** hepatectomy, laparoscopic liver resection, liver neoplasms, robotic liver resection, treatment outcome

## Abstract

**Objective:**

To compare the perioperative outcomes of robotic liver surgery (RLS) and laparoscopic liver surgery (LLS) in various settings.

**Background:**

Clear advantages of RLS over LLS have rarely been demonstrated, and the associated costs of robotic surgery are generally higher than those of laparoscopic surgery. Therefore, the exact role of the robotic approach in minimally invasive liver surgery remains to be defined.

**Methods:**

In this international retrospective cohort study, the outcomes of patients who underwent RLS and LLS for all indications between 2009 and 2021 in 34 hepatobiliary referral centers were compared. Subgroup analyses were performed to compare both approaches across several types of procedures: (1) minor resections in the anterolateral (2, 3, 4b, 5, and 6) or (2) posterosuperior segments (1, 4a, 7, 8), and (3) major resections (≥3 contiguous segments). Propensity score matching was used to mitigate the influence of selection bias. The primary outcome was textbook outcome in liver surgery (TOLS), previously defined as the absence of intraoperative incidents ≥grade 2, postoperative bile leak ≥grade B, severe morbidity, readmission, and 90-day or in-hospital mortality with the presence of an R0 resection margin in case of malignancy. The absence of a prolonged length of stay was added to define TOLS+.

**Results:**

Among the 10.075 included patients, 1.507 underwent RLS and 8.568 LLS. After propensity score matching, both groups constituted 1.505 patients. RLS was associated with higher rates of TOLS (78.3% vs 71.8%, *P* < 0.001) and TOLS+ (55% vs 50.4%, *P* = 0.026), less Pringle usage (39.1% vs 47.1%, *P* < 0.001), blood loss (100 vs 200 milliliters, *P* < 0.001), transfusions (4.9% vs 7.9%, *P* = 0.003), conversions (2.7% vs 8.8%, *P* < 0.001), overall morbidity (19.3% vs 25.7%, *P* < 0.001), and microscopically irradical resection margins (10.1% vs. 13.8%, *P* = 0.015), and shorter operative times (190 vs 210 minutes, *P* = 0.015). In the subgroups, RLS tended to have higher TOLS rates, compared with LLS, for minor resections in the posterosuperior segments (n = 431 per group, 75.9% vs 71.2%, *P* = 0.184) and major resections (n = 321 per group, 72.9% vs 67.5%, *P* = 0.086), although these differences did not reach statistical significance.

**Conclusions:**

While both produce excellent outcomes, RLS might facilitate slightly higher TOLS rates than LLS.

In light of surgeons’ pursuit of less invasive treatment modalities, with the aim of improving clinical outcomes, minimally invasive surgery has gained traction over the past decades. However, for liver surgery, the uptake of the minimally invasive approach has been rather slow, due to concerns about hemorrhage control, oncological safety, and the long learning curve of minimally invasive liver surgery (MILS).^1^ Despite these initial challenges, pioneering surgeons working in highly specialized centers have refined their MILS techniques and reported favorable outcomes in selected patients.^2,3^

After these early experiences, international guidelines and a plethora of observational and randomized studies have appraised the efficacy of MILS.^1,4–9^ Hence, the minimally invasive approach has become the reference approach for many liver surgical procedures in expert centers.^1,10^ Originally, MILS was mainly performed using the laparoscopic approach, but more recently the robotic approach has been increasingly adopted.^11,12^ In theory, robotic liver surgery (RLS) should offer at least comparable benefits over open surgery as laparoscopic liver surgery (LLS), and recent studies have supported this hypothesis.^13,14^ Nevertheless, evidence supporting the implementation of RLS is still relatively scarce, and its associated costs are generally higher than those of laparoscopic surgery.^11^ In fact, clear advantages of RLS over LLS have rarely been demonstrated, despite the technical advantages that the robot offers, such as integrated 3-dimensional systems, improved stability, and dexterity.^15–17^

Therefore, the exact role of the robotic approach in liver surgery remains to be defined. The aim of this study is thus to compare the perioperative outcomes of RLS and LLS in various settings. The composite outcome measure “textbook outcome” was used as the primary outcome measure, as composite outcome measures may offer a more accurate reflection of overall surgical quality.^18,19^

## METHODS

### Study Design

To perform this international multicenter retrospective cohort study, the prospectively maintained databases of 34 hepatobiliary referral centers from 15 countries were bundled and retrospectively assessed. Consecutive patients (≥18 years) who underwent an elective robotic or laparoscopic liver resection from January 2009 to December 2021 were included. Patients who underwent hand-assisted procedures, preoperative portal vein embolization, portal vein ligation or associating liver partition and portal vein ligation for staged hepatectomy, major concurrent procedures (eg, vascular or biliary reconstructions, colorectal, diaphragmatic, or pancreatic resections) and patients who did not undergo a formal liver resection (eg, cyst fenestration) were excluded. The included patients were stratified according to the allocated surgical approach (robotic or laparoscopic). Thereafter, subgroups were created according to the type of procedure that was performed: (1) minor resections in the anterolateral segments (segments 2, 3, 4b, 5, and 6), (2) posterosuperior segments (segments 1, 4a, 7, 8), and (3) major resections (3 or more contiguous Couinaud segments). The characteristics and perioperative outcomes of RLS and LLS in the overall cohort and subgroups were compared before and after propensity score matching (PSM), which was applied to mitigate the influence of selection bias.^20,21^ A standardized survey was conducted among the participating robotic surgeons to clarify whether they regularly use the Cavitron Ultrasonic Surgical Aspirator (CUSA, Integra LifeSciences Corporation), operated by the bedside surgeon. The survey question was formulated as follows, specified for laparoscopic and robotic surgery separately: “How do you perform liver parenchymal transection in your center? Do you use Energy devices, CUSA, or both?” The medical ethical committee of Brescia approved this study and waived the need to obtain informed consent due to its retrospective nature and the use of pseudonymized data. (judgment’s reference number: NP 5403) This report was written following the guidelines outlined in the “Strengthening The Reporting of Observational Studies in Epidemiology” statement.^22^

### Definitions and Outcomes

Data were collated from electronic health records. Baseline characteristics comprised patient demographics, American Society of Anesthesiologists score, presence of cirrhosis and if present Child-Pugh scale, treatment with neoadjuvant chemotherapy, history of extrahepatic or hepatic abdominal surgery, disease characteristics (type, number of lesions, size of the largest lesion, and uni or bilobar distribution) and the extent and type of resection performed. The extent of liver resections was defined according to the Brisbane 2000 terminology.^23^ In addition, the “New World Terminology” equivalents, as described by Nagino et al,^24^ were added between brackets to non–self-explanatory definitions of resections (ie, right hepatectomy, right posterior sectionectomy, etc). No standardized terminology is available for the term “segmentectomy,” but this was considered the resection of the majority of a Couinaud segment. A bisegmentectomy and trisegmentectomy were considered the resection of the majority of 2 or 3 contiguous segments, respectively. A resection of 3 or more contiguous segments was defined as major. Minor resections in the anterolateral or posterosuperior segments were separately reported and analyzed, due to the increased technical difficulty of minimally invasive resections in the posterosuperior segments.^5,25^ The Institut Mutualiste Montsouris difficulty score was assigned to each laparoscopic and robotic resection, defined according to Kawaguchi et al,^26^ as follows: grade 1 includes wedge resection and left lateral sectionectomy, grade 2 includes anterolateral segmentectomy and left hepatectomy (H234), and grade 3 includes posterosuperior segmentectomy, right posterior sectionectomy (H67), right hepatectomy (H5678), central hepatectomy (H458), and extended left/right hepatectomy (H23458, H45678, respectively). The intraoperative outcomes included operative time in minutes, estimated blood loss in milliliters, usage and duration of the Pringle maneuver, perioperative packed red blood cell transfusion, intraoperative unfavorable incidents, and conversion to an open procedure. The postoperative outcomes consisted of length of stay, morbidity, and readmissions at 30 days and 90 day or in-hospital mortality. The Oslo classification was used to define and grade intraoperative unfavorable incidents. Postoperative morbidity was defined and graded using the Clavien-Dindo classification and reported as overall and severe (Clavien-Dindo: ≥ 3a).^27,28^ Posthepatectomy bile leak and liver failure were defined and graded according to their respective International Study Group of Liver Surgery classifications.^29,30^ Whether or not a patient achieved a textbook outcome was derived from the available perioperative outcome data. The validated survey-based definition of textbook outcome in liver surgery (TOLS) was used.^31^ Thus, TOLS was defined as the absence of intraoperative incidents of grade 2 or higher, postoperative bile leak grade B or C, severe morbidity, readmission, and 90-day or in-hospital mortality with the presence of an R0 resection margin in case of malignancy. The absence of a prolonged length of stay was added to define textbook outcome + (TOLS+), using the previously reported cut-offs of >4 days for minor and >7 days for major resections.^31^

### Statistical Analyses

Several variables contained missing data in a missing at-random pattern (Supplemental Digital Content Fig. 1, http://links.lww.com/SLA/F45). Therefore, a single imputation was applied. Outcome data were not imputed. Categorical data were reported as counts and percentages and compared between the robotic and laparoscopic groups using χ^2^ or Fisher exact test, when appropriate. Normally distributed continuous data were reported as the mean with its standard deviation (SD) and compared using an unpaired *T* test. Non-normally distributed continuous data were reported as the median with its range and compared using the Mann-Whitney *U* test. The distribution was evaluated by visual inspection of histograms and Q-Q plots. Subsequently, PSM was applied in a 1:1 ratio without replacement on the overall cohort and the predefined procedure subgroups, using a caliper width of 0.2.^32^ Propensity scores were calculated using multivariable logistic regression models.^33^ Factors that could influence the allocation to robotic or laparoscopic surgery were entered as covariates in this model: age, sex, American Society of Anesthesiologists classification, presence of cirrhosis and grade (Child-Pugh Scale), history of previous hepatic surgery, type of resection, and type and extent of disease (pathologic diagnosis, number of lesions, size of the largest lesions, and uni or bilobar distribution). A sensitivity analysis was conducted, wherein this process was repeated on the subgroup of patients that underwent surgery from January 2015 onwards, to correct for possible influences of the learning curve and improvements in perioperative care. After matching, a balance was assessed using standardized differences. A SD ≤0.1 is considered optimal balance.^34^ Categorical data were compared using the McNemar test. Ordinal and continuous data were compared using the Wilcoxon signed-rank test. All analyses were performed according to the intention-to-treat principle. A 2-sided *P* value < 0.05 was considered statistically significant. Data were analyzed using IBM SPSS Statistics version 29.0 (IBM) and R for Mac OS X version 4.2.1 (R Foundation for Statistical Computing).

## RESULTS

**FIGURE 1:**
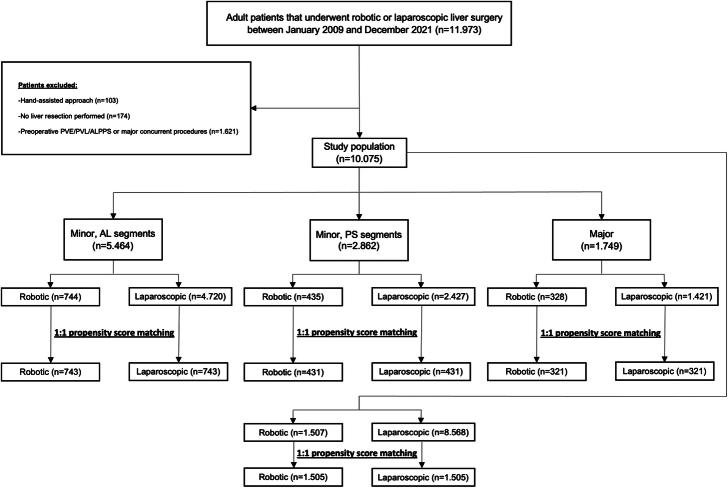
Study flowchart. AL, indicate anterolateral; ALPPS, associating liver partition and portal vein ligation for staged hepatectomy; PS, posterosuperior; PVE, portal vein embolization; PVL, portal vein ligation.

Overall, 10.075 patients were included (Fig. 1). Of these patients, 1.507 underwent RLS and 8.568 LLS. Of the participating centers, 23 centers performed both RLS and LLS, 9 centers only LLS, and 2 centers only RLS. The subgroups comprised 5.464 patients for minor resections in the anterolateral segments, 2.862 patients for minor resections in the posterosuperior segments, and 1.749 patients for major resections.

### Patient Characteristics and Perioperative Outcomes in the Overall Cohort, Before Propensity Score Matching

Baseline characteristics of patients allocated to RLS and LLS revealed that the RLS group was associated with slightly younger age (Median 62 vs 64.6 years, *P* < 0.001) and a higher prevalence of liver cirrhosis (25% vs 20.4%, *P* < 0.001). In terms of lesion characteristics, the RLS group was associated with more singular lesions {median: 1 [interquartile range (IQR): 1–1] vs 1 (IQR: 1–2), *P* < 0.001}, and larger lesion size (median 36 vs 30 mm, *P* < 0.001). Furthermore, while a greater proportion of patients in the RLS group was affected by hepatocellular carcinoma (34.3% vs 25.8%, *P* < 0.001) or benign liver disease (27% vs 17.7%, *P* < 0.001), the proportion of patients with colorectal liver metastases was significantly lower (21.6% vs 40.1%, *P* < 0.001; Supplemental Digital Content Table 1, http://links.lww.com/SLA/F45). The RLS group demonstrated lower rates of previous hepatic surgery (5.7% vs 9.3%, *P* < 0.001) and treatment with neoadjuvant chemotherapy (16.7% vs 26.7%, *P* < 0.001). Concerning the performed procedures, the proportion of major resections was higher in the RLS group (21.9% vs 16.6%, *P* < 0.001), whereas the proportion of patients who underwent concurrent thermal ablations was lower (2.2% vs 5%, *P* < 0.001). Compared with LLS, the RLS group generally consisted of patients that underwent resections with higher Institut Mutualiste Montsouris difficulty scores (grade 1: 72.4% vs 62.3%, grade 2: 15.2% vs 23.3%, grade 3: 12.4% vs 14.4%, *P* < 0.001).

Intraoperatively, RLS was associated with a longer operative time [median: 190 (IQR: 139–272) vs 190 minutes (123–270), *P* = 0.013], a shorter Pringle duration when applied (median: 30 vs 40 minutes, *P* < 0.001), less blood loss (median: 100 vs 200 mL, *P* < 0.001), transfusions (4.9% vs 6.2%, *P* = 0.046), grade 2 intraoperative incidents (2.4% vs 4.4%, *P* < 0.001), and conversions (2.7% vs 7.1%, *P* < 0.001; Table 1). During the postoperative course, the median length of stay was 4 days in both the patients allocated to RLS and LLS (*P* = 0.008). RLS was, however, associated with slightly lower rates of microscopically positive resection margins (R1; 10.1% vs 15%, *P* < 0.001), paralleled by higher rates of TOLS (78.3% vs 71.8%, *P* < 0.001) and TOLS+ (54.9% vs 50.9%, *P* = 0.005). Conversely, the readmission rate was higher in the robotic group (6.3% vs 4.4%, *P* = 0.002).

**TABLE 1:** Intra and Postoperative Outcomes in the Overall Cohort Stratified by the Used Surgical Approach, Before and After PSM

	Before PSM			After PSM		
	Robotic (n = 1.507)	Laparoscopic (n = 8.568)	*P*	Robotic (n = 1.505)	Laparoscopic (n = 1.505)	*P*
Intraoperative						
Pringle maneuver	589 (39.2)	3450 (41.3)	0.118	587 (39.1)	692 (47.1)	<0.001
Pringle duration	30 (20, 45)	40 (25, 60)	<0.001	30 (20, 45)	40 (25, 60)	<0.001
Operative time	190 (139, 272)	190 (123, 270)	0.013	190 (139, 272)	210 (136.3, 300)	0.015
Intraoperative blood loss	100 (50, 280)	200 (100, 400)	<0.001	100 (50, 280)	200 (100, 400)	<0.001
Transfusion of packed cells	72 (4.9)	468 (6.2)	0.046	72 (4.9)	105 (7.9)	0.003
No. of transfusions	2 (1, 3)	2 (1, 3)	0.595	2 (1, 3)	2 (1, 3)	0.850
Intraoperative incidents	—	—	<0.001	—	—	0.003
Grade 1	129 (8.7)	427 (5.6)	—	129 (8.7)	86 (6.4)	—
Grade 2	36 (2.4)	338 (4.4)	—	36 (2.4)	77 (5.7)	—
Grade 3	3 (0.2)	10 (0.1)	—	3 (0.2)	2 (0.1)	—
Conversion	39 (2.7)	591 (7.1)	<0.001	39 (2.7)	130 (8.8)	<0.001
Postoperative						
Length of stay, days	4 (3, 6)	4 (3, 6)	0.008	4 (3, 6)	4 (3, 6)	0.398
Overall morbidity	291 (19.3)	1830 (21.5)	0.060	291 (19.3)	384 (25.7)	<0.001
Severe morbidity	97 (6.4)	593 (7.0)	0.465	97 (6.5)	113 (7.6)	0.331
Readmission	93 (6.3)	337 (4.4)	0.002	93 (6.3)	66 (4.9)	0.090
90-day or in-hospital mortality	23 (1.5)	113 (1.3)	0.511	23 (1.5)	21 (1.4)	0.880
Resection margin status	—	—	<0.001	—	—	0.015
Microscopically radical (R0)	1130 (89.8)	6546 (84.7)	—	1129 (89.8)	1126 (86)	—
Microscopically irradical (R1)	127 (10.1)	1160 (15.0)	—	127 (10.1)	180 (13.8)	—
Macroscopically irradical (R2)	1 (0.1)	20 (0.3)	—	1 (0.1)	3 (0.2)	—
Prolonged length of stay*	486 (32.5)	2661 (31.7)	0.530	484 (32.4)	501 (33.9)	0.493
Textbook outcome	1093 (78.3)	5275 (71.8)	<0.001	1091 (78.3)	941 (71.8)	<0.001
Textbook outcome +	779 (54.9)	3918 (50.9)	0.005	779 (55)	689 (50.4)	0.026

Values are expressed in counts (percentages) or in median (IQR).

Counts may not add up due to missing data.

* Defined as >4 days for minor and >7 days for major liver resections.

### Patient Characteristics and Perioperative Outcomes in the Overall Cohort, After Propensity Score Matching

After PSM, the RLS and LLS groups both included 1.505 patients. Optimal balance between the groups, with respect to the preselected covariates, was observed after matching (All SD ≤ 0.055; Supplemental Digital Content Table 1, http://links.lww.com/SLA/F45). Concerning intraoperative outcomes, RLS was now associated with less Pringle usage (31.9% vs 47.1%, *P* < 0.001), shorter operative times (190 vs 210 minutes, *P*=0.015), a shorter Pringle duration (median: 30 vs 40 minutes, *P* < 0.001), less blood loss (100 vs 200 mL, *P* < 0.001), transfusions (4.9% vs 7.9%, *P* = 0.003), grade 2 intraoperative incidents (2.4% vs 5.7%, *P* = 0.003), and conversions (2.7% vs 8.8%, *P* < 0.001; Table 1). Postoperatively, RLS was associated with reduced rates of overall morbidity (19.3% vs 25.7%, *P* < 0.001), R1 resections (10.1% vs 13.8%, *P* = 0.015), increased rates of achieving TOLS (78.3% vs 71.8%, *P* < 0.001), and TOLS+ (55% vs 50.4%, *P* = 0.026).

### Perioperative Outcomes in the Subgroup of Minor Resections in the Anterolateral Segments, After Propensity Score Matching

The subgroup of patients who underwent a minor resection in the anterolateral segments consisted of 744 patients allocated to RLS and 4.720 patients allocated to LLS. After PSM, 743 patients remained in each group. The included covariates were well balanced after matching (All SD ≤ 0.052; Supplemental Digital Content Table 2, http://links.lww.com/SLA/F45). Intraoperatively, RLS offered several benefits over LLS, in terms of less Pringle usage (26.5% vs 34.2%, *P* < 0.001), shorter Pringle duration when applied (median: 25 vs 33.5 minutes, *P* = 0.023), less blood loss (median: 100 vs 150 mL, *P* < 0.001), transfusions (2.6% vs 5.5%, *P* = 0.010), and conversions (1.2% vs 4.8%, *P* < 0.001; Table 2). Despite observing slightly higher rates of R0 resection margins, TOLS, and TOLS+ readmissions, these differences were not statistically significant.

**TABLE 2:** Intra and Postoperative Outcomes of Minor Resections in the Anterolateral Segments Stratified by the Used Surgical Approach, After PSM

	Robotic (n = 743)	Laparoscopic (n = 743)	*P*
Intraoperative			
Pringle maneuver	196 (26.5)	247 (34.2)	<0.001
Pringle duration	25 (18.3, 37.8)	33.5 (20, 50)	0.023
Operative time	165 (120, 225)	160 (110, 235)	0.394
Intraoperative blood loss	100 (30, 200)	150 (50, 300)	<0.001
Transfusion of packed cells	19 (2.6)	36 (5.5)	0.010
No. of transfusions	2 (1, 3)	2 (1, 3)	NA
Intraoperative incidents			0.439
Grade 1	64 (8.8)	26 (3.9)	—
Grade 2	9 (1.2)	15 (2.2)	—
Grade 3	1 (0.1)	3 (0.4)	—
Conversion	9 (1.2)	35 (4.8)	<0.001
Postoperative			
Length of stay (d)	3.9 (2, 5)	4 (2, 6)	0.362
Overall morbidity	144 (19.4)	153 (20.8)	0.558
Severe morbidity	39 (5.3)	42 (5.7)	0.822
Readmission	39 (5.3)	28 (4.1)	0.314
90 d or in-hospital mortality	14 (1.9)	9 (1.2)	0.383
Resection margin status			0.124
Microscopically radical (R0)	536 (89.8)	549 (87.6)	—
Microscopically irradical (R1)	60 (10.1)	77 (12.3)	—
Macroscopically irradical (R2)	1 (0.2)	1 (0.2)	—
Prolonged length of stay*	227 (30.7)	250 (34.4)	0.130
Textbook outcome	564 (82)	508 (79.1)	0.452
Textbook outcome +	410 (58.5)	358 (53.5)	0.069

Values are expressed in counts (percentages) or in median (IQR).

Counts may not add up due to missing data.

* Defined as >4 days.

NA indicates not available.

### Perioperative Outcomes in the Subgroup of Minor Resections in the Posterosuperior Segments, After Propensity Score Matching

In the subgroup of patients who underwent a minor resection in the posterosuperior segments, 435 patients were allocated to RLS and 2.427 patients to LLS. After PSM, both groups consisted of 431 patients. The chosen covariates were well-balanced after matching (Supplemental Digital Content Table 2, http://links.lww.com/SLA/F45). In these patients, RLS was associated with a shorter Pringle duration (median: 30 vs 45 minutes, *P* = 0.011), less blood loss (median: 100 vs 200 mL, *P* < 0.001), and a lower conversion rate (2.9% vs 10.9%, *P* < 0.001). In addition, RLS achieved higher rates of R0 resection margins (88.3% vs 85.1%, *P* = 0.104) and TOLS (75.9% vs 71.2%, *P* = 0.184), although not reaching statistical significance (Table 3).

**TABLE 3:** Intra and Postoperative Outcomes of Minor Resections in the Posterosuperior Segments Stratified by the Used Surgical Approach, After PSM

	Robotic (n = 431)	Laparoscopic (n = 431)	*P*
Intraoperative			
Pringle maneuver	227 (52.8)	230 (53.9)	0.884
Pringle duration	30 (20, 45)	45 (25, 69.5)	<0.001
Operative time	192 (150, 270)	210 (140, 300)	0.144
Intraoperative blood loss	100 (50, 280)	200 (100, 400)	<0.001
Transfusion of packed cells	26 (6.1)	25 (6.5)	1
No. of transfusions	2 (1, 3)	2 (1, 3)	1
Intraoperative incidents			0.278
Grade 1	44 (10.5)	31 (7.9)	—
Grade 2	11 (2.6)	21 (5.3)	—
Grade 3	0	1 (0.3)	—
Conversion	12 (2.9)	45 (10.9)	<0.001
Postoperative			
Length of stay (d)	4 (3, 5.8)	4 (3, 6)	0.584
Overall morbidity	80 (18.6)	96 (22.3)	0.218
Severe morbidity	28 (6.5)	24 (5.6)	0.677
Readmission	24 (5.8)	19 (5.0)	0.749
90 d or in-hospital mortality	3 (0.7)	6 (1.4)	0.505
Resection margin status			0.104
Microscopically radical (R0)	331 (88.3)	315 (85.1)	—
Microscopically irradical (R1)	44 (11.7)	53 (14.3)	—
Macroscopically irradical (R2)	0	2 (0.5)	—
Prolonged length of stay*	168 (39.6)	176 (42.5)	0.375
Textbook outcome	296 (75.9)	262 (71.2)	0.184
Textbook outcome +	189 (47.2)	170 (44)	0.303

Values are expressed in counts (percentages) or in median (IQR).

Counts may not add up due to missing data.

* Defined as >4 days.

### Perioperative Outcomes in the Subgroup of Major Resections, After Propensity Score Matching

Of the patients who underwent a major resection, 328 patients were allocated to RLS and 1.421 patients to LLS. After PSM, 321 adequately matched patients remained in each group (all SD ≤ 0.084; Supplemental Digital Content Table 3, http://links.lww.com/SLA/F45). Intraoperatively, RLS was associated with less Pringle usage (49.1% vs 60%, *P* < 0.001), reduced blood loss (median: 190 vs 300 mL, *P* < 0.001), and lower conversion rates (5.4% vs 10.3%, *P* = 0.027). Postoperatively, RLS was associated with a lower overall morbidity rate (20.6% vs 33.8%, *P* < 0.001), and tended to achieve higher TOLS rates (72.9% vs 67.5%, *P* = 0.086; Table 4).

**TABLE 4:** Intra and Postoperative Outcomes of Major Resections Stratified by the Used Surgical Approach, After PSM

	Robotic (n = 321)	Laparoscopic (n = 321)	*P*
Intraoperative			
Pringle maneuver	157 (49.1)	189 (60)	<0.001
Pringle duration	30 (20, 50)	43 (30, 57)	0.049
Operative time	270 (200, 366)	300 (240, 370)	0.197
Intraoperative blood loss	190 (50, 400)	300 (200, 527.5)	<0.001
Transfusion of packed cells	26 (8.2)	25 (8.8)	0.760
No. of transfusions	2 (1.3, 2)	2 (1, 4)	NA
Intraoperative incidents			0.128
Grade 1	20 (6.3)	25 (8.9)	—
Grade 2	16 (5)	18 (6.4)	—
Grade 3	2 (0.6)	0	—
Conversion	17 (5.4)	33 (10.3)	0.027
Postoperative			
Length of stay (d)	5 (4, 8)	5 (4, 7.3)	0.748
Overall morbidity	66 (20.6)	108 (33.8)	<0.001
Severe morbidity	30 (9.3)	43 (13.4)	0.154
Readmission	28 (8.9)	12 (4.5)	0.201
90-day or in-hospital mortality	5 (1.6)	4 (1.3)	1
Resection margin status			0.401
Microscopically radical (R0)	254 (91.7)	266 (89.9)	—
Microscopically irradical (R1)	23 (8.3)	30 (10.1)	—
Macroscopically irradical (R2)	0	0	—
Prolonged length of stay*	82 (25.7)	81 (25.5)	1
Textbook outcome	223 (72.9)	179 (67.5)	0.086
Textbook outcome +	177 (57.8)	154 (55)	0.562

Values are expressed in counts (percentages) or in median (IQR).

Counts may not add up due to missing data.

* Defined as >7 days.

NA indicates not available.

### Sensitivity Analysis of the Procedures Performed From January 2015 Onwards

In the sensitivity analysis, wherein both approaches were compared in the time period from January 2015 onwards, 1.394 patients who underwent RLS were adequately matched to 1.394 patients who underwent LLS (Supplemental Digital Content Table 4, http://links.lww.com/SLA/F45). This analysis largely demonstrated comparable benefits of RLS over LLS, and a similar higher rate of TOLS with RLS (79.9% vs 72.5%, *P* = 0.001; Table 5).

**TABLE 5:** Intra and Postoperative Outcomes in the Overall Cohort From 2015 Onwards Stratified by the Used Surgical Approach, After PSM

	Robotic (n = 1.394)	Laparoscopic (n = 1.394)	*P*
Intraoperative			
Pringle maneuver	550 (39.6)	679 (49.7)	<0.001
Pringle duration	29 (20, 45)	40 (25, 62)	<0.001
Operative time	190 (136, 270)	210 (134.3, 299.5)	0.023
Intraoperative blood loss	100 (50, 250)	200 (100, 400)	<0.001
Perioperative blood transfusions	65 (4.8)	100 (8.0)	<0.001
No. of transfusions	2 (1, 2.3)	2 (1, 3)	1
Intraoperative incidents			0.027
Grade 1	121 (8.8)	91 (7.3)	—
Grade 2	34 (2.5)	64 (5.2)	—
Grade 3	3 (0.2)	2 (0.2)	—
Conversion	35 (2.6)	112 (8.2)	<0.001
Postoperative			
Length of stay (d)	4 (3, 6)	4 (3, 6)	0.868
Overall morbidity	260 (18.7)	341 (24.6)	<0.001
Severe morbidity	84 (6.0)	111 (8.0)	0.047
Readmission	89 (6.5)	61 (4.9)	0.036
90 d or in-hospital mortality	20 (1.5)	24 (1.7)	0.651
Resection margin status			0.002
Microscopically radical (R0)	1056 (90.1)	1048 (86)	—
Microscopically irradical (R1)	116 (9.9)	168 (13.8)	—
Macroscopically irradical (R2)	0	3 (0.2)	—
Prolonged length of stay*	452 (32.7)	448 (32.7)	1
Textbook outcome	1021 (78.9)	885 (72.5)	0.001
Textbook outcome +	724 (55.1)	651 (51.3)	0.165

Values are expressed in counts (percentages) or in median (IQR).

Counts may not add up due to missing data.

* Defined as >4 days for minor and >7 days for major liver resections.

### Survey on the Use of Cavitron Ultrasonic Surgical Aspirator in Robotic Liver Surgery

Of the 25 participating centers that perform RLS, 24 centers responded (response rate 96%). Five of these centers use CUSA regularly during robotic liver resections (21%). The survey revealed that the decision to use CUSA during robotic liver resection is based on the type and extent of the planned resection, and the surgeon’s preference.

## DISCUSSION

This large international multicenter cohort study, in which the perioperative outcomes of RLS versus LLS for all indications were compared, identified several benefits of RLS. In the overall PSM cohort, RLS was associated with lower rates of Pringle usage and shorter Pringle duration, less blood loss, transfusions, and conversions. In addition, RLS was associated with lower postoperative morbidity rates, whereas a larger proportion of the patients after RLS were readmitted. Finally, RLS was associated with higher rates of TOLS and TOLS +.

While the robotic approach is increasingly adopted in the field of liver surgery, evidence supporting this trend remains limited. In this context, relying solely on the analysis of individual outcomes for perioperative assessment may result in an inaccurate representation of the overall situation. Textbook outcome amalgamates several intra and postoperative outcomes into a single variable, effectively representing the most favorable outcome after a surgical procedure.^35^ In recent years, textbook outcome measures have gained traction across various surgical specialties, and the achievement of textbook outcomes has been linked to increased survival in esophagogastric and pancreatic surgery.^36,37^ Gorgec et al^31^ defined, based on an international survey among hepatobiliary surgeons, and validated TOLS, thus providing a potent outcome assessment tool in this domain. Of note, the addition of the variable “absence of a prolonged length of stay” (named TOLS +) did not reach the 80% consensus threshold in this definition, which, therefore, requires a more nuanced interpretation. Our analysis generally revealed higher TOLS rates in the RLS group, especially in the subgroups of minor resections in the posterosuperior segments and major resections, indicating a potential benefit of RLS in this setting. These findings are also in line with the expectation that the enhanced dexterity and superior visual capabilities of the robotic approach could mainly be beneficial during more complex resections.^15,38^

Despite the ongoing debate on its advantages and disadvantages, the Pringle maneuver has been increasingly employed in recent decades to reduce blood loss during parenchymal transection and thus facilitate a dry surgical field.^39,40^ In our analysis, we consistently found lower Pringle usage and shorter duration in the RLS group, which is in line with earlier reports.^41–43^ Nevertheless, RLS was associated with slightly less blood loss and lower transfusion rates. In a meta-analysis by Gavriilidis et al,^13^ both RLS and LLS were associated with comparable intraoperative amounts of blood loss, but more recent reports also support the marginal benefits of RLS, with regard to blood loss and transfusion, observed in this study.^43–45^ These findings indicate that, despite the absence of CUSA (Integra LifeSciences Corporation) in the robotic toolkit, the robotic approach may offer a greater degree of bleeding control. One possible explanation for this could be the aforementioned stable surgical field, which facilitates improved visualization and allows for a more controlled and safer dissection of the vasculo-biliary structures within the liver parenchyma and at the hepatic hilum. In addition, the higher performance of the wrist-like articulating robotic hook and bipolar instruments may play a role, as they offer the advantage of more precise application of electrical force. Although PSM was applied, another contributing factor could be the disease characteristics of patients allocated to RLS, as patients with less extensive disease are often selected in the early implementation phase of a new technique.

In this study, the conversion rates were markedly lower when the robotic approach was used (2.7% vs 8.8%, *P* < 0.001). In initial reports, the conversion rates seemed to be comparable for both the laparoscopic and robotic approaches.^13,46^ Currently, however, the properties of the robot seem to offer certain benefits in this regard, which allow surgeons to complete more procedures in a minimally invasive manner.^15^ Some authors have suggested that the decrease in conversion rates is related to the fact that there is less need to convert to achieve oncological radicality and control bleeding in RLS.^16,43^ The exact reason for this difference, however, remains unclear, warranting additional studies focusing on this topic. Conversions, especially when in an emergency setting, have been associated with inferior postoperative outcomes.^47^ The lower conversion rates of the robotic group could thus result in better postoperative outcomes. Nevertheless, the postoperative outcomes of both groups were generally comparable, although RLS was associated with a slightly lower overall morbidity but a higher readmission rate. Interestingly, an earlier multicenter study with a smaller sample size even associated LLS with a lower overall morbidity rate.^16^

The baseline characteristics of the unmatched cohort suggest that the robotic approach is more often adopted for technically complex cases, such as minor resections in the posterosuperior segments or major resections, implying a certain degree of patient selection. The large sample size of this cohort allowed us to perform several subgroup analyses, gaining more insight into the possible merits of the robotic approach in specific surgical settings. In these subgroup analyses, the robotic approach was not associated with a statistically significant benefit in terms of TOLS rates in any of the subgroups. When comparing individual perioperative outcomes, our findings are consistent with the results of several other studies. The modest reduction in intraoperative blood loss and the lower conversion rates in robotic minor resections in the anterolateral segments mirror those found by Kadam et al^45^ in their matched analysis. A study by D’Silva et al,^44^ comparing outcomes of robotic and laparoscopic minor resections in the posterosuperior segments, found a comparably lower Pringle duration, less intraoperative blood loss, and lower conversion rates as the present study. A study by Liu et al,^43^ which focused on major liver resections, found less intraoperative blood loss, Pringle application, and lower conversion rates, similar to our subgroup analysis of major liver resections. Their study also found a significant difference in length of hospital stay (6 vs 7 days), which in the present study was equal in both groups (5 days), possibly owing to national extramural health care differences between the participating centers. An interesting area for future research would also be the assessment of the efficacy of RLS in specific patient populations, such as patients affected by obesity or cirrhosis, as the absence of the CUSA in RLS might especially lead to difficulties during parenchymal transection in patients with chronic liver disease and cirrhosis.^48,49^ It would also be interesting to compare overall morbidity rates after RLS and LLS using the Comprehensive Complication Index.^50^ Unfortunately, the multicenter database that was used to perform this study lacks the granularity to reliably calculate this index.

This study has several limitations that need to be acknowledged and discussed. First, its retrospective and observational design can lead to loss of data and at least a certain degree of selection bias. Although PSM was used to mitigate the influence of selection bias, a side effect of this statistical technique is that ultimately treatment effects are compared between subgroups of the entire cohort.^51^ Furthermore, this approach fails to consider any unknown confounding factors. Second, the learning curve might have had an effect on the witnessed outcomes. To address this, we conducted a sensitivity analysis including only procedures performed in the last half of the study period, which yielded results that were consistent with those observed in the overall cohort. Nevertheless, the observed results might still differ from the contemporary situation in expert centers with extensive experience in LLS and/or RLS. Third, surgical techniques and perioperative care are likely to differ, to a certain degree, between participating centers, reflecting the variability that is present in daily clinical practice. This includes the performed surgical technique and used instruments in anatomic liver resection. The aim of this study was, however, to report on the present-day practices and perioperative outcomes in a large number of hepatobiliary centers across the world.

## CONCLUSIONS

While both RLS and LLS produce excellent outcomes when adopted to perform minor and major liver resections in selected patients, the robotic approach might facilitate slightly higher textbook outcome rates than laparoscopy. These findings should be confirmed in well-designed randomized studies comparing RLS and LLS in specific surgical settings.

## Supplementary Material
