# Supplementary material for: Robotic Versus Laparoscopic Liver Resection in Various Settings: An International Multicenter Propensity Score Matched Study of 10.075 Patients
Source: Ann Surg. 2024 Mar 14;280(1):108–17. doi: 10.1097/SLA.0000000000006267 (PMC11161239; doi:10.1097/SLA.0000000000006267)
Supplement: Supplementary file 1 [file sla-280-108-s001.docx]

| **Supplementary table 1.** Baseline, disease and procedural characteristics in the overall cohort stratified by the used surgical approach, before and after propensity score matching | | | | | | | |
| --- | --- | --- | --- | --- | --- | --- | --- |
|  | **Before PSM** | | | **After PSM** | | | |
|  | **Robotic** | **Laparoscopic** | **P** | **Robotic** | **Laparoscopic** | **P** | **SD** |
|  | **n = 1.507** | **n = 8.568** |  | **n = 1.505** | **n = 1.505** |  |  |
| Age (years) | 62 [52, 70] | 64.6 [55, 72.3] | <0.001 | 62 [52, 70] | 62 [51, 71] | 0.450 | 0.040 |
| BMI | 25.5 [23.1, 29.3] | 25.7 [23, 29] | 0.047 | 25.5 [23.1, 29.3] | 25.9 [22.8, 29] | 0.110 | 0.055 |
| Gender, male | 855 (56.7) | 4818 (56.2) | 0.717 | 853 (56.7) | 869 (57.7) | 0.584 | 0.021 |
| ASA-score ≥ 3 | 550 (36.5) | 3237 (37.8) | 0.343 | 550 (36.5) | 547 (36.3) | 0.940 | 0.004 |
| Cirrhosis & Child-Pugh scale |  |  | <0.001 |  |  | 0.933 | 0.025 |
| No cirrhosis | 1130 (75) | 6821 (79.6) |  | 1130 (75.1) | 1130 (75.1) |  |  |
| Child-Pugh A | 302 (20) | 1616 (18.9) |  | 302 (20.1) | 305 (20.3) |  |  |
| Child-Pugh B | 74 (4.9) | 129 (1.5) |  | 72 (4.8) | 68 (4.5) |  |  |
| Child-Pugh C | 1 (0.1) | 2 (0.0) |  | 1 (0.1) | 2 (0.1) |  |  |
| Neoadjuvant chemotherapy | 252 (16.7) | 2287 (26.7) | <0.001 | 252 (16.7) | 258 (17.1) | 0.795 | 0.011 |
| Previous abdominal surgery |  |  |  |  |  |  |  |
| Extrahepatic | 658 (43.7) | 3603 (42.1) | 0.243 | 658 (43.7) | 495 (32.9) | <0.001 | 0.224 |
| Hepatic | 86 (5.7) | 793 (9.3) | <0.001 | 86 (5.7) | 103 (6.8) | 0.221 | 0.047 |
| Disease |  |  | <0.001 |  |  | 0.615 | 0.046 |
| Colorectal metastases | 326 (21.6) | 3434 (40.1) |  | 326 (21.7) | 307 (20.4) |  |  |
| Hepatocellular carcinoma | 517 (34.3) | 2209 (25.8) |  | 516 (34.3) | 511 (34) |  |  |
| Cholangiocarcinoma | 97 (6.4) | 454 (5.3) |  | 97 (6.4) | 91 (6) |  |  |
| Gallbladder carcinoma | 45 (3) | 112 (1.3) |  | 45 (3) | 49 (3.3) |  |  |
| Benign | 407 (27) | 1517 (17.7) |  | 406 (27) | 428 (28.4) |  |  |
| Non colorectal metastases | 81 (5.4) | 672 (7.8) |  | 81 (5.4) | 84 (5.6) |  |  |
| Other malignancy | 34 (2.3) | 170 (2) |  | 34 (2.3) | 35 (2.3) |  |  |
| Number of lesions | 1 [1, 1] | 1 [1, 2] | <0.001 | 1 [1, 1] | 1 [1, 1] | 0.018 | 0.055 |
| Bilobar distribution | 181 (12) | 1154 (13.5) | 0.124 | 181 (12) | 181 (12) | 1 | <0.001 |
| Size largest lesion, millimeters | 36 [22, 60] | 30 [18, 50] | <0.001 | 36 [22, 60] | 35 [20, 60] | 0.719 | 0.003 |
| Extent of resection |  |  | <0.001 |  |  | 0.956 | 0.011 |
| Minor - anterolateral segments | 744 (49.4) | 4720 (55.1) |  | 744 (49.4) | 751 (49.9) |  |  |
| Wedge | 275 (18.2) | 2486 (29) |  | 275 (18.3) | 278 (18.5) |  |  |
| Segmentectomy | 195 (12.9) | 775 (9) |  | 195 (13) | 191 (12.7) |  |  |
| Bisegmentectomy | 274 (18.2) | 1459 (17) |  | 274 (18.2) | 282 (18.7) |  |  |
| Minor - posterosuperior segments | 435 (28.9) | 2427 (28.3) |  | 433 (28.8) | 426 (28.3) |  |  |
| Wedge | 188 (12.5) | 1445 (16.9) |  | 188 (12.5) | 192 (12.8) |  |  |
| Segmentectomy | 124 (8.2) | 438 (5.1) |  | 122 (8.1) | 115 (7.7) |  |  |
| Bisegmentectomy | 123 (8.2) | 544 (6.4) |  | 123 (8.2) | 119 (7.9) |  |  |
| Major | 328 (21.8) | 1421 (16.6) |  | 328 (21.8) | 328 (21.8) |  |  |
| Trisegmentectomy | 53 (3.5) | 157 (1.8) |  | 53 (3.5) | 48 (3.2) |  |  |
| Hemihepatectomy | 237 (15.7) | 1121 (13.1) |  | 237 (15.7) | 251 (16.7) |  |  |
| Extended hemihepatectomy | 27 (1.8) | 98 (1.1) |  | 27 (1.8) | 22 (1.5) |  |  |
| Central hepatectomy | 11 (0.7) | 42 (0.5) |  | 11 (0.7) | 7 (0.5) |  |  |
| Other | 0 | 3 (0.0) |  | 0 | 0 |  |  |
| IMM classification |  |  | <0.001 |  |  | 0.655 | 0.066 |
| Grade I | 939 (62.3) | 6199 (72.4) |  | 939 (62.4) | 948 (63) |  |  |
| Grade II | 351 (23.3) | 1299 (15.2) |  | 349 (23.2) | 314 (20.9) |  |  |
| Grade III | 217 (14.4) | 1061 (12.4) |  | 217 (14.4) | 242 (16.1) |  |  |
| Type of resection |  |  | <0.001 |  |  | 0.006 | 0.117 |
| Non-anatomical | 606 (40.2) | 4160 (48.6) |  | 606 (40.3) | 534 (35.5) |  |  |
| Anatomical | 826 (54.8) | 3836 (44.8) |  | 824 (54.8) | 867 (57.6) |  |  |
| Combined anatomical/non-anatomical | 75 (5) | 572 (6.7) |  | 75 (5) | 104 (6.9) |  |  |
| Concurrent resection | 374 (24.8) | 1954 (22.8) | 0.088 | 374 (24.9) | 347 (23.1) | 0.261 | 0.042 |
| Concurrent thermal ablation | 33 (2.2) | 429 (5) | <0.001 | 33 (2.2) | 70 (4.7) | <0.001 | 0.136 |
| Values are expressed in percentages or in median [IQR].  Abbreviations: BMI, body mass index; ASA, American Society of Anaesthesiologists.  Counts may not add up due to missing data. | | | | | | | |

| **Supplementary table 2.** Baseline, disease and procedural characteristics in subgroups of minor resections in the AL and PS segments, stratified by the used surgical approach, after propensity score matching | | | | | | | | |
| --- | --- | --- | --- | --- | --- | --- | --- | --- |
|  | **Minor AL segments** | | | | **Minor PS segments** | | | |
|  | **Robotic** | **Laparoscopic** | **P** | **SD** | **Robotic** | **Laparoscopic** | **P** | **SD** |
|  | **n = 743** | **n = 743** |  |  | **n = 431** | **n = 431** |  |  |
| Age (years) | 61.2 [52, 70] | 62.8 [52, 71] | 0.465 | 0.018 | 62 [52.6, 70.6] | 63 [51.7, 71] | 0.659 | 0.043 |
| BMI | 26.3 [23.4, 30.1] | 26 [23.3, 29.5] | 0.173 | 0.069 | 25.2 [23, 28.8] | 25.2 [22.6, 29] | 0.583 | 0.017 |
| Gender, male | 385 (51.8) | 370 (49.8) | 0.460 | 0.040 | 264 (61.3) | 272 (63.1) | 0.602 | 0.038 |
| ASA-score ≥ 3 | 296 (39.8) | 294 (39.6) | 0.956 | 0.006 | 152 (35.3) | 158 (36.7) | 0.711 | 0.029 |
| Cirrhosis & Child-Pugh scale |  |  | 0.724 | 0.019 |  |  | 0.822 | 0.024 |
| No cirrhosis | 553 (74.4) | 557 (75.0) |  |  | 320 (74.2) | 319 (74) |  |  |
| Child-Pugh A | 150 (20.2) | 149 (20.1) |  |  | 95 (22) | 94 (21.8) |  |  |
| Child-Pugh B | 40 (5.4) | 37 (5) |  |  | 16 (3.7) | 18 (4.2) |  |  |
| Child-Pugh C | 0 | 0 |  |  |  |  |  |  |
| Neoadjuvant chemotherapy | 103 (13.9) | 94 (12.7) | 0.521 | 0.036 | 82 (19) | 99 (23) | 0.146 | 0.097 |
| Previous abdominal surgery |  |  |  |  |  |  |  |  |
| Extrahepatic | 318 (42.8) | 269 (36.2) | 0.010 | 0.135 | 194 (45) | 164 (38.1) | 0.027 | 0.142 |
| Hepatic | 47 (6.3) | 38 (5.1) | 0.386 | 0.052 | 27 (6.3) | 28 (6.5) | 1 | 0.009 |
| Disease |  |  | 0.984 | 0.033 |  |  | 0.494 | 0.082 |
| Colorectal metastases | 132 (17.8) | 134 (18) |  |  | 122 (28.3) | 129 (29.9) |  |  |
| Hepatocellular carcinoma | 240 (32.3) | 233 (31.4) |  |  | 166 (38.5) | 164 (38.1) |  |  |
| Cholangiocarcinoma | 50 (6.7) | 48 (6.5) |  |  | 15 (3.5) | 13 (3) |  |  |
| Gallbladder carcinoma | 44 (5.9) | 47 (6.3) |  |  | 1 (0.2) | 0 |  |  |
| Benign | 223 (30) | 229 (30.8) |  |  | 89 (20.6) | 88 (20.4) |  |  |
| Non colorectal metastases | 33 (4.4) | 31 (4.2) |  |  | 31 (7.2) | 31 (7.2) |  |  |
| Other malignancy | 21 (2.8) | 21 (2.8) |  |  | 7 (1.6) | 6 (1.4) |  |  |
| Number of lesions | 1 [1, 1] | 1 [1, 1] | 0.174 | 0.028 | 1 [1, 1] | 1 [1, 2] | 0.355 | 0.049 |
| Bilobar distribution | 67 (9.0) | 76 (10.2) | 0.460 | 0.041 | 53 (12.3) | 49 (11.4) | 0.740 | 0.029 |
| Size largest lesion, millimeters | 35 [20, 55] | 35 [20, 55] | 0.667 | 0.003 | 33 [21, 50] | 30 [20, 50] | 0.381 | 0.001 |
| Extent of resection |  |  | 0.552 | 0.047 |  |  | 0.743 | 0.048 |
| Wedge | 275 (37) | 280 (37.7) |  |  | 188 (43.6) | 180 (41.8) |  |  |
| Segmentectomy | 194 (26.1) | 205 (27.6) |  |  | 120 (27.8) | 129 (29.9) |  |  |
| Bisegmentectomy | 274 (36.9) | 258 (34.7) |  |  | 123 (28.5) | 122 (28.3) |  |  |
| IMM classification |  |  | NA | <0.001 |  |  | 0.074 | 0.164 |
| Grade I | 743 (100) | 743 (100) |  |  | 188 (43.6) | 181 (42) |  |  |
| Grade II | 0 | 0 |  |  | 196 (45.5) | 179 (41.5) |  |  |
| Grade III | 0 | 0 |  |  | 47 (10.9) | 71 (16.5) |  |  |
| Type of resection |  |  | 0.073 | 0.119 |  |  | 0.002 | 0.210 |
| Non-anatomical | 359 (48.3) | 318 (42.8) |  |  | 227 (52.7) | 193 (44.8) |  |  |
| Anatomical | 368 (49.5) | 402 (54.1) |  |  | 179 (41.5) | 192 (44.5) |  |  |
| Combined anatomical/non-anatomical | 16 (2.2) | 23 (3.1) |  |  | 25 (5.8) | 46 (10.7) |  |  |
| Concurrent resection | 185 (24.9) | 149 (20.1) | 0.024 | 0.116 | 92 (21.3) | 104 (24.1) | 0.349 | 0.066 |
| Concurrent thermal ablation | 11 (1.5) | 27 (3.6) | 0.015 | 0.137 | 20 (4.6) | 26 (6.0) | 0.461 | 0.062 |
| Values are expressed in percentages or in median [IQR].  Abbreviations: BMI, body mass index; ASA, American Society of Anaesthesiologists.  Counts may not add up due to missing data. | | | | | | | | |

| **Supplementary table 3.** Baseline, disease and procedural characteristics in the subgroup of major resections, stratified by the used surgical approach, after propensity score matching | | | | |
| --- | --- | --- | --- | --- |
|  | **Robotic** | **Laparoscopic** | **P** | **SD** |
|  | **n = 321** | **n = 321** |  |  |
| Age (years) | 62 [52, 70] | 62 [50, 72] | 0.529 | 0.051 |
| BMI | 25 [22.9, 28.1] | 25.2 [22.5, 28.1] | 0.504 | 0.087 |
| Gender, male | 195 (60.7) | 191 (59.5) | 0.793 | 0.025 |
| ASA-score ≥ 3 | 101 (31.5) | 98 (30.5) | 0.863 | 0.020 |
| Cirrhosis & Child-Pugh scale |  |  | 0.598 | 0.068 |
| No cirrhosis | 255 (79.4) | 262 (81.6) |  |  |
| Child-Pugh A | 57 (17.8) | 49 (15.3) |  |  |
| Child-Pugh B | 9 (2.8) | 10 (3.1) |  |  |
| Neoadjuvant chemotherapy | 67 (20.9) | 74 (23.1) | 0.547 | 0.053 |
| Previous abdominal surgery |  |  |  |  |
| Extrahepatic | 143 (44.5) | 101 (31.5) | <0.001 | 0.272 |
| Hepatic | 12 (3.7) | 14 (4.4) | 0.831 | 0.032 |
| Disease |  |  | 0.997 | 0.084 |
| Colorectal metastases | 72 (22.4) | 76 (23.7) |  |  |
| Hepatocellular carcinoma | 103 (32.1) | 102 (31.8) |  |  |
| Cholangiocarcinoma | 31 (9.7) | 24 (7.5) |  |  |
| Gallbladder carcinoma | 0 | 0 |  |  |
| Benign | 92 (28.7) | 94 (29.3) |  |  |
| Non colorectal metastases | 17 (5.3) | 18 (5.6) |  |  |
| Other malignancy | 6 (1.9) | 7 (2.2) |  |  |
| Number of lesions | 1 [1, 1] | 1 [1, 2] | 0.961 | 0.040 |
| Bilobar distribution | 61 (19) | 65 (20.2) | 0.766 | 0.031 |
| Size largest lesion, millimeters | 53 [30, 80] | 51 [28, 82] | 0.981 | 0.024 |
| Extent of resection |  |  | 0.665 | 0.047 |
| Trisegmentectomy | 51 (15.9) | 46 (14.3) |  |  |
| Hemihepatectomy | 232 (72.3) | 235 (73.2) |  |  |
| Extended hemihepatectomy | 27 (8.4) | 29 (9.0) |  |  |
| Central hepatectomy | 11 (3.4) | 11 (3.4) |  |  |
| IMM classification |  |  | 0.943 | 0.007 |
| Grade I | 7 (2.2) | 7 (2.2) |  |  |
| Grade II | 146 (45.5) | 144 (45.1) |  |  |
| Grade III | 168 (52.3) | 168 (52.7) |  |  |
| Type of resection |  |  | <0.001 | 0.322 |
| Non-anatomical | 18 (5.6) | 2 (0.6) |  |  |
| Anatomical | 269 (83.8) | 296 (92.2) |  |  |
| Combined anatomical/non-anatomical | 34 (10.6) | 23 (7.2) |  |  |
| Concurrent resection | 95 (29.6) | 72 (22.4) | 0.049 | 0.164 |
| Concurrent thermal ablation | 2 (0.6) | 6 (1.9) | 0.289 | 0.113 |
| Values are expressed in percentages or in median [IQR].  Abbreviations: BMI, body mass index; ASA, American Society of Anaesthesiologists.  Counts may not add up due to missing data. | | | | |

| **Supplementary table 4.** Baseline, disease and procedural characteristics in the cohort from 2015 onwards stratified by the used surgical approach, after propensity score matching | | | | |
| --- | --- | --- | --- | --- |
|  | **Robotic** | **Laparoscopic** | **P** | **SD** |
|  | **n = 1.394** | **n = 1.394** |  |  |
| Age (years) | 62 [52.1, 70] | 63 [52.1, 72] | 0.364 | 0.030 |
| BMI | 25.5 [23.1, 29.2] | 25.7 [23, 29] | 0.365 | 0.043 |
| Gender, male | 804 (57.7) | 799 (57.3) | 0.880 | 0.007 |
| ASA-score ≥ 3 | 506 (36.3) | 533 (38.2) | 0.301 | 0.040 |
| Cirrhosis & Child-Pugh scale |  |  | 0.662 | 0.027 |
| No cirrhosis | 1037 (74.4) | 1034 (74.2) |  |  |
| Child-Pugh A | 288 (20.7) | 283 (20.3) |  |  |
| Child-Pugh B | 68 (4.9) | 76 (5.5) |  |  |
| Child-Pugh C | 1 (0.1) | 1 (0.1) |  |  |
| Neoadjuvant chemotherapy | 229 (16.4) | 260 (18.7) | 0.112 | 0.058 |
| Previous abdominal surgery |  |  |  |  |
| Extrahepatic | 601 (43.1) | 456 (32.7) | <0.001 | 0.216 |
| Hepatic | 82 (5.9) | 80 (5.7) | 0.932 | 0.006 |
| Disease |  |  | 0.715 | 0.064 |
| Colorectal metastases | 296 (21.2) | 288 (20.7) |  |  |
| Hepatocellular carcinoma | 490 (35.2) | 499 (35.8) |  |  |
| Cholangiocarcinoma | 89 (6.4) | 72 (5.2) |  |  |
| Gallbladder carcinoma | 40 (2.9) | 42 (3) |  |  |
| Benign | 371 (26.6) | 378 (27.1) |  |  |
| Non colorectal metastases | 78 (5.6) | 88 (6.3) |  |  |
| Other malignancy | 30 (2.2) | 27 (1.9) |  |  |
| Number of lesions | 1 [1, 1] | 1 [1, 1] | 0.045 | 0.060 |
| Bilobar distribution | 168 (12.1) | 182 (13.1) | 0.444 | 0.030 |
| Size largest lesion, millimeters | 35 [22, 60] | 35 [20, 60] | 0.978 | 0.013 |
| Extent of resection |  |  | 0.781 | 0.039 |
| Minor - anterolateral segments | 666 (47.8) | 677 (48.6) |  |  |
| Wedge | 251 (18) | 276 (19.8) |  |  |
| Segmentectomy | 173 (12.4) | 174 (12.5) |  |  |
| Bisegmentectomy | 242 (17.4) | 227 (16.3) |  |  |
| Minor - posterosuperior segments | 407 (29.2) | 383 (27.5) |  |  |
| Wedge | 174 (12.5) | 155 (11.1) |  |  |
| Segmentectomy | 117 (8.4) | 115 (8.2) |  |  |
| Bisegmentectomy | 116 (8.3) | 113 (8.1) |  |  |
| Major | 321 (23) | 334 (24) |  |  |
| Trisegmentectomy | 51 (3.7) | 52 (3.7) |  |  |
| Hemihepatectomy | 234 (16.8) | 244 (17.5) |  |  |
| Extended hemihepatectomy | 25 (1.8) | 27 (1.9) |  |  |
| Central hepatectomy | 11 (0.8) | 11 (0.8) |  |  |
| Other | 0 | 0 |  |  |
| IMM classification |  |  | 0.351 | 0.055 |
| Grade I | 847 (60.8) | 840 (60.3) |  |  |
| Grade II | 337 (24.2) | 318 (22.8) |  |  |
| Grade III | 210 (15.1) | 236 (16.9) |  |  |
| Type of resection |  |  | 0.003 | 0.135 |
| Nonanatomical | 560 (40.2) | 496 (35.6) |  |  |
| Anatomical | 763 (54.7) | 788 (56.5) |  |  |
| Combined anatomical/nonanatomical | 71 (5.1) | 110 (7.9) |  |  |
| Concurrent resection | 341 (24.5) | 334 (24) | 0.787 | 0.012 |
| Concurrent thermal ablation | 28 (2) | 80 (5.7) | <0.001 | 0.194 |
| Values are expressed in percentages or in median [IQR].  Abbreviations: BMI, body mass index; ASA, American Society of Anaesthesiologists.  Counts may not add up due to missing data. | | | | |


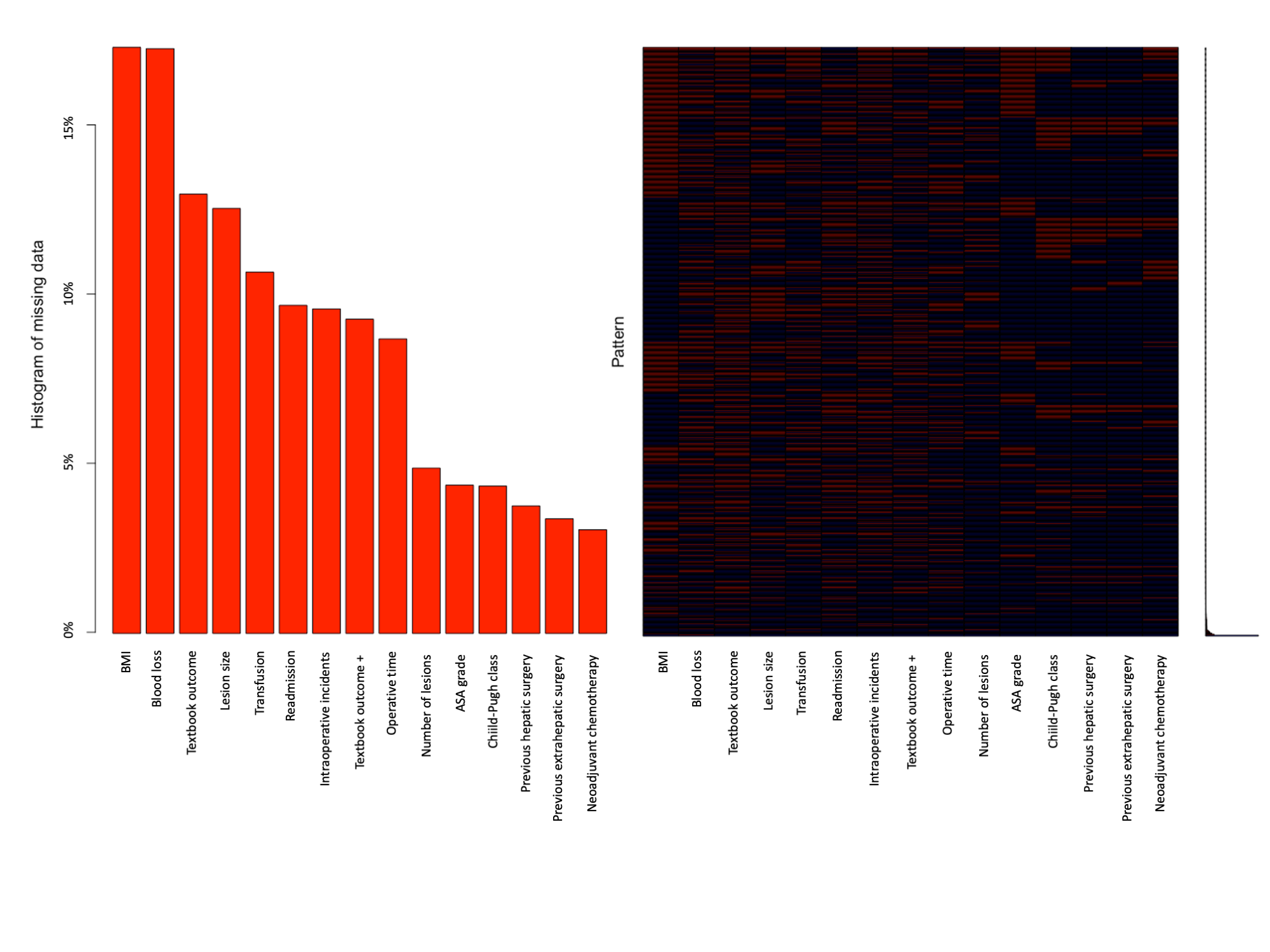
**Supplementary figure 1.** Overview of missing data (for variables with more than 3% missing data)
